# Recruitment, adherence and attrition challenges in internet-based indicated prevention programs for eating disorders: lessons learned from a randomised controlled trial of *ProYouth OZ*

**DOI:** 10.1186/s40337-021-00520-7

**Published:** 2022-01-04

**Authors:** Kathina Ali, Daniel B. Fassnacht, Louise M. Farrer, Elizabeth Rieger, Markus Moessner, Stephanie Bauer, Kathleen M. Griffiths

**Affiliations:** 1 Research School of Psychology, The Australian National University, Canberra, Australia; 2 Centre for Mental Health Research, The Australian National University, Canberra, Australia; 3 College of Education, Psychology and Social Work, Flinders University, Adelaide, Australia; 4 Órama Institute for Mental Health and Wellbeing, Adelaide, Australia; 5 Centre for Psychotherapy Research, University Hospital Heidelberg, Heidelberg, Germany

**Keywords:** Online intervention, Peer support, Mental health, Barriers, Stigma, Eating disorder literacy, Help-seeking, Body image, Digital health

## Abstract

**Background:**

Growing evidence supports the effectiveness of Internet-based prevention programs for eating disorders, but the adjunctive benefit of synchronous peer support has yet to be investigated. In the current study, a randomised controlled trial was conducted to evaluate the effectiveness of an indicated Internet-based prevention program (*ProYouth OZ)* with and without peer-to-peer support in reducing disordered eating behaviours and attitudes.

**Method:**

Fifty young adults (18–25 years) with eating disorder symptoms were randomised to one of three study conditions: (1) *ProYouth OZ* (without peer-to-peer support), (2) *ProYouth OZ Peers* (with peer-to-peer support), and (3) a waitlist control group. Outcomes were assessed at three different time points. Eating disorder symptoms (primary outcome) were measured with the Eating Disorder Examination Questionnaire.

**Results:**

Of 415 screened participants, 73 (17.6%) were eligible and 213 (51.3%) excluded due to severe eating disorder symptoms. Fifteen participants (30%) completed the post-intervention survey. Of the two intervention groups, 20.6% failed to access any component of the program. Of 17 *ProYouth OZ Peers* participants, 58.8% attended at least one chat session, 20% attended 2–5 sessions, and 11.8% attended all six sessions. Due to limited outcome data, it was not possible to statistically examine between-group differences in outcomes. Visual inspection of individual profiles revealed that both *ProYouth OZ Peers* participants who completed the post-intervention survey showed a decrease in disordered eating compared with only one of the six completers in *ProYouth OZ*.

**Conclusion:**

Findings highlight the challenges of trialling Internet-based eating disorder prevention programs in the community. The study identified a large group of emerging adults with eating disorders who were interested in an Internet-based program, suggesting a high level of unmet need. Future research on synchronous peer-to-peer support in Internet-based prevention for eating disorders is warranted. Further studies are required to identify optimal strategies for reaching this population (e.g., online vs. offline) and evaluating the effectiveness of a range of strategies for promoting engagement. Finally, there is an urgent need to develop innovative widely accessible interventions for individuals who experience clinically relevant eating disorder symptomatology but may not be ready or able to seek professional face-to-face treatment.

*Trial registration*: ACTRN12615001250527, Registered 16 November 2015, https://www.anzctr.org.au/Trial/Registration/TrialReview.aspx?ACTRN=12615001250527

**Supplementary Information:**

The online version contains supplementary material available at 10.1186/s40337-021-00520-7.

## Plain English summary

Internet-based prevention programs for eating disorders are effective, but a variety of challenges have been found when offering them in real-world settings. The current study aimed to test *ProYouth OZ,* an Internet-based eating disorder prevention program consisting of multiple components including peer support. In total, 50 young adults (18–25 years) with eating disorder symptoms were allocated to one of three versions of the program. In total, 21% did not access the program. From 17 participants in the peer support group, 60% attended at least one chat session and 12% attended all six sessions. Given that only fifteen participants (30%) completed the questionnaire after the intervention, it is not possible to draw reliable conclusions about the effectiveness of the program. Findings demonstrate the difficulty of recruiting emerging adults, engaging them with the program and collecting data after participation. The majority of interested participants experienced an eating disorder, suggesting they may be searching for help online. Future studies should investigate the use of peer support, identify strategies for reaching eligible participants and develop approaches to increase engagement. Finally, it is important to develop innovative programs that provide self-help strategies and facilitate help-seeking for those who are experiencing an eating disorder.

**Supplementary Information:**

The online version contains supplementary material available at 10.1186/s40337-021-00520-7.

## Background

The onset of eating disorders most often occurs during adolescence and early adulthood [1–4]. Biological, social and psychological risk factors have been identified for the development of these serious mental illnesses which are associated with limited quality of life, psychiatric comorbidity, chronicity, increased mortality risk, and high relapse rates [5–9]. Despite the severe psychological and physical impairment associated with eating disorders, only a minority of individuals seek and receive professional help [10]. Reasons underlying low help-seeking rates include stigma and shame, minimising the severity of the illness, low motivation to change, negative attitudes towards seeking help and treatment, lack of encouragement from others to seek help, lack of knowledge about help resources, and practical barriers including financial and transport issues [11–14]. Internet-based interventions have the potential to overcome some of these barriers by offering anonymity, privacy, accessibility, availability, and cost-effectiveness, and may be perceived as less stigmatising than face-to-face treatments [15–18]. Moreover, given its potential to deliver interventions to a broad range of individuals, the Internet holds particular promise as a platform for delivering programs to prevent eating disorders.

A variety of Internet-based programs for eating disorders have been developed with overall evidence suggesting that they offer a promising approach to preventing and treating eating disorders [19–24]. While several studies have investigated Internet-based programs targeting young females with eating disorder symptoms [22, 23], only five studies have undertaken randomised controlled trials of indicated (i.e., applied to individuals who exhibit elevated symptoms of eating disorders without meeting diagnostic criteria for the disorder) Internet-based eating disorder prevention programs. Four involved different versions of the cognitive behavioural therapy-based prevention program *Student Bodies* [25–28], one of the most widely tested prevention programs in the field of eating disorders. The fifth study investigated an Internet-based version of the dissonance-based prevention program *The Body Project* [29]. With the exception of the study by Vollert et al. [28] who focussed on the impact of recruitment strategies and did not report outcomes, all trials reported eating disorder risk reduction following the intervention [25–27, 29]. However, all studies recruited only young females and mainly university students, with only two recruiting a very small percentage of members of the community [25, 28], thus limiting the external validity of findings. In addition, all studies used face-to-face assessments and two used external rewards (i.e., monetary compensation) [27, 29] which may prove difficult to implement in real-world settings.

The majority of Internet-based programs for eating disorders consist of multiple components, including psychoeducation, self-monitoring, and therapist or peer-to-peer support [15, 19]. Online peer-to-peer support (or peer support) provides a platform for participants to share experiences, and to seek, receive, and provide information, advice, and emotional support [30]. Communication between peers can occur via asynchronous (e.g., forums, bulletin boards, and discussion groups) and synchronous platforms (e.g., chat groups). However, evidence for the effect of online peer-to-peer support on eating disorders is sparse and inconsistent. Although one study of *Student Bodies* with and without asynchronous peer discussion groups found that peer support did not enhance weight, shape or eating-related outcomes [31], a subsequent randomised controlled trial [32] demonstrated that participants who received the intervention with asynchronous peer discussion groups showed a significantly greater reduction in weight and shape concerns than those without access to the discussion group. These two studies involved moderated asynchronous peer support; however, to date no randomised controlled trial has investigated the effects of synchronous peer support (e.g., online peer chat groups) in an Internet-based prevention program for eating disorders.

In addition to outcome studies, research is needed to identify strategies for improving adherence (i.e., program usage) and attrition (i.e., failure to complete assessments) in Internet-based programs. High dropout rates (i.e., not starting or finishing the intervention or not completing assessments) are commonly reported in trials of these programs with one systematic review of Internet-based interventions for eating disorders reporting dropouts ranging from 9 to 47.2% [21]. One approach that might increase participant retention is to offer choice in the material or program components participants can access. The Internet-based prevention program for eating disorders, *ProYouth,* allows users to access the amount and type of intervention content that best meets their level of symptomatology or clinical need [15]. Originally developed in Germany and implemented in nine European countries, there is evidence of its acceptability and effectiveness [33–37], and its potential to facilitate access to traditional care [38, 39].

### Rationale and study objectives

The original objective of the current trial was to investigate the effectiveness of an adapted version of *ProYouth (ProYouth OZ)* with and without synchronous peer-to-peer support with respect to disordered eating behaviours and attitudes relative to each other and a waitlist control group. In addition to describing the trial protocol and the participant flow and characteristics, this paper aims to convey the challenges encountered with recruitment, adherence and attrition as well as lessons learned from the trial as a basis for providing recommendations for future research.

## Method

### Study design

The study was a three-arm randomised controlled trial (RCT) designed to evaluate the effectiveness of online peer-to-peer support in the Internet-based prevention program *ProYouth OZ*. Young adults at risk for eating disorders were randomised into *ProYouth OZ*, *ProYouth OZ Peers* or a waitlist control condition. Primary and secondary outcomes were measured at pre- and post-intervention (6 weeks), and at 3- and 6-month follow-up (after post-intervention). Ethics approval was obtained from the Australian National University Human Ethics Research Committee (protocol number 2015/742). The trial was registered with the Australian and New Zealand Clinical Trials Registry (ACTRN12615001250527).

### Participant recruitment

Recruitment occurred over a 12-month period from October 2016 and was open to all Australian residents aged 18–25 years. The study was advertised at two major universities in the Australian Capital Territory, via print and on social media and through Australian mental health organisations including the National Eating Disorders Collaboration, the Butterfly Foundation, and Headspace. Further, the study was promoted by emerging adults with a lived experience of an eating disorder via various social media channels (i.e., Facebook, YouTube, Tumblr, Twitter, and Instagram). In addition, a Facebook community page with access to the screening survey was created to raise awareness about the study and engage potential participants.

Interested participants accessed a link that directed them to the study website hosted at the Australian National University or to the *ProYouth OZ* Facebook page. After providing consent to participate, individuals completed a brief 5-min screening survey to assess eligibility. Those who did not meet inclusion criteria due to experiencing severe symptoms of an eating disorder, received feedback incorporating various resources encouraging them to seek professional help. Those who met inclusion criteria were asked to provide a valid email address so that they could be sent an invitation to complete the pre-intervention assessment. Detailed information about the study procedure was provided before participants’ informed consent was obtained electronically.

### Inclusion/exclusion criteria

Only participants at risk for an eating disorder were eligible for participation in the trial, according to the following criteria: (1) a score greater than 57 on the Weight Concerns Scale (WCS) (based on previous research findings that 10% of females in the highest quartile of the WCS develop subclinical or full eating disorders) [40, 41], and/or (2) recurrent binge eating, and/or compensatory behaviours at a frequency of less than once a week or for a duration of less than 3 months assessed with the Short Evaluation of Eating Disorders (SEED). Additional inclusion criteria were: age (18–25 years), willingness and ability to access the Internet, a valid email address, and consent to participate.

Consistent with the definition of indicated prevention and in contrast to *ProYouth* in Germany, the current study excluded participants who met criteria for an eating disorder. The following criteria were used to identify and exclude those potentially fulfilling criteria for a clinical eating disorder: (1) current eating disorder diagnosis, or (2) a BMI of less than 17, or (3) binge eating/vomiting/use of laxatives at a frequency of more than once a week or once a week for more than 3 months, or (4) current eating disorder treatment. Further exclusion criteria were: (5) vision or reading problems, or (6) self-reported diagnosis of an eating disorder, substance-related disorder, post-traumatic stress disorder, schizophrenia, or personality disorder.

### Sample size

The sample size was based on an anticipated small to medium interaction effect (group by time) of Cohen’s *f* = 0.15 in the primary outcome (i.e., disordered eating behaviours and attitudes). Assuming an alpha of 0.05, a power of 0.80, a medium correlation of *r* = 0.3 between pre- and post-intervention, and accounting for 30% dropout, a sample size of 201 (67 per group) was required to compare the three groups from pre- to post-intervention (GPower [42]).

### Randomisation procedure

Participants were randomised to one of the three study conditions after having completed the pre-intervention assessment. The randomisation was conducted manually by an independent researcher not involved in the delivery of the intervention using a randomisation schedule created with a random number generator. Stratified randomisation was used to minimise differences in gender distributions.

### Study conditions

The study involved three conditions: (1) *ProYouth OZ*, (2) *ProYouth OZ Peers*, and (3) a waitlist control group.

#### ProYouth OZ

Participants allocated to the *ProYouth OZ* condition were provided with access to the Internet-based prevention program for a duration of 6 weeks. *ProYouth OZ* was adapted from the European program *ProYouth* for use in Australia. The program was revised to target young adults at high risk for developing an eating disorder, namely, individuals with elevated weight and shape concerns and early symptoms without meeting criteria for a threshold eating disorder. The *ProYouth OZ* program comprises psychoeducational information and a monitoring and feedback system. The different components of the program aim to educate and raise awareness, help individuals to detect problematic attitudes and behaviour, reduce help-seeking barriers, and facilitate help-seeking for those in need. The psychoeducation section included written information about the different characteristics and possible diagnostic indicators of eating disorders, general information about health and wellbeing, prevention and self-help, fact sheets, and help-seeking resources. This section of the program was adapted from the original *ProYouth* program from Germany. For example, *ProYouth OZ* featured personal stories written by young adults from Australia who had recovered from an eating disorder.

Participants also had access to the monitoring and feedback system which was designed to assess eating disorder-related symptoms, enable the early detection of symptom elevation, and provide minimal intervention using supportive feedback. As part of the monitoring and feedback system, participants received 6 weekly invitations to complete eight questions assessing cognitive and behavioural aspects of the following four dimensions: body dissatisfaction, overconcern with weight and shape, poor nutrition/dieting, and binge eating and compensatory behaviours. Participants responded on a four-point Likert scale and received automatically generated feedback messages. These messages were based on the participant’s reported change in eating disorder related attitudes and behaviours and were rated as (1) *improved* (from dysfunctional to functional), (2) *deteriorated* (from functional to dysfunctional), (3) *unchanged* (functional) or (4) *unchanged* (dysfunctional). The messages were intended to motivate and encourage participants to maintain positive eating-related attitudes and behaviours, to provide support, and to reinforce positive changes. Given the patterns of change on these four dimensions of eating disorder symptoms, there were 256 possible feedback categories. Each category included several statements which were adapted from a pool of existing messages from previous research on the original *ProYouth* program. An algorithm selected a different message each week precluding repeated messages if participants’ responses did not change over time. An example message is: “*Your well-balanced eating patterns are great. However, you seem to be worried about your body. Try not to let thoughts about shape and weight take over. We all have times we wish that our bodies looked different. Think about your favourite people in your life and what you value most about them. Chances are, it’s not their weight or body shape. Remember that others feel the same about you. Think about three qualities you have that others value.*”

#### ProYouth OZ Peers

Participants allocated to the *ProYouth OZ Peers* condition gained for a duration of 6 weeks access to the aforementioned components of the *ProYouth OZ* program, as well as to an online peer-to-peer support component. Participants were encouraged to attend weekly 1-h chat sessions led by a peer with a lived experience of an eating disorder in the presence of a trained health professional with more than 5 years’ experience in moderating online chat groups. Groups consisted of four to six participants and were intended to provide a safe and supportive environment to facilitate discussions around body image and eating concerns. In contrast to other Internet-based interventions that included the use of chat groups to deliver cognitive-behaviour therapy content [43–45], the groups in the current study were primarily led by the peer in recovery who facilitated the discussion by sharing her own experiences and learning opportunities, whereas the main role of the trained health professional was to ensure a safe environment. The chat sessions aimed to enable participants and the peer facilitator to encourage and support each other and identify similar distress and body-related experiences. Prior to entering the chat group, participants were required to agree to the chat guidelines, which, for example, did not allow people to share their weight as this may have been triggering for some participants and further stated that the discussion of unhealthy behaviours would not be tolerated.

The topics for each chat session were developed based on discussions with emerging adults who had recovered from an eating disorder and feedback from clinical psychologists and experts in the field of eating disorders. The first chat session provided participants with the opportunity to introduce themselves, tell their story and explore their reasons for participation. The second session facilitated a discussion around positive body image, while the third session focused on envisioning how life would be without body image struggles. The fourth session discussed coping strategies, and the fifth session examined help-seeking pathways and the experience of seeking help. The final session allowed participants to summarise their learning experience (e.g., new skills and strategies) and focused on key take home messages.

At the beginning of each session, participants were invited to watch a brief video of peers who have recovered from an eating disorder which was intended to provide messages of hope, insight, and a sense of belonging to participants. Evidence suggests that peer videos may have the potential to reduce stigma by encouraging a discussion about concerns associated with stigma and shame [46], a topic that was addressed as part of the videos. In addition, the inclusion of videos has been noted as a means of rendering a prevention program more engaging [47].

#### Waitlist control group

Participants in the waitlist control group were asked to complete the pre-, post- and 3-month follow-up assessments, after which they were provided with access to *ProYouth OZ*.

### Procedure

Blinding of the participant’s group allocation was not possible due to the nature of the study. Participants in the *ProYouth OZ* condition were provided with immediate access to the program. Once sufficient participants for a chat group (i.e., four to six participants) had been randomly allocated to the *ProYouth OZ Peers* condition, each was sent an email inviting them to access the program. Participants in both conditions were sent weekly email reminders to complete the monitoring, and, at the end of the 6-week intervention period, an email invitation to complete the post-intervention assessment. In addition, participants were advised by email of follow-up surveys at 3 and 6 months after completion of the intervention. If participants failed to complete the survey, they were sent three additional reminders via email.

At any stage of the study, participants could withdraw from the trial without explanation by emailing the trial manager. After the two groups had completed the 6-week intervention and the 3-month follow-up, the same procedure as described for the *ProYouth OZ* condition was followed for those who had been randomised to the waitlist control group.

### Measures

All outcomes were assessed using self-report, online questionnaires at pre-, post-intervention, and at two follow-ups 3 and 6 months after the intervention (see Additional file 1). Sociodemographic variables were assessed at baseline.

#### Sociodemographic variables

Sociodemographic variables were assessed at baseline and included gender, age, ethnicity, country of birth, language, main daily activities, education level, student status, internet usage, and self-reported Body Mass Index (BMI = kg/m^2^).

#### Screening measures

##### Weight and shape concerns

The Weight Concerns Scale (WCS; [40, 41]) is a five-item screening questionnaire that assesses fear of weight gain, worry about weight and body shape, the importance of weight, diet history, and perceived fatness. Scores range from 0 to 100, with higher scores indicating greater weight concerns. Previous studies have shown that 10% of girls in the highest quartile of the WCS (> 57) develop subclinical or full eating disorders [40, 41]. The questionnaire demonstrates good test–retest reliability and predictive validity [40, 41].

##### Eating disorder symptomatology

The Short Evaluation of Eating Disorders (SEED; [48]) is a six-item screening instrument for eating disorder symptomatology. It assesses core characteristics of eating disorders (e.g., the frequency of binge eating and compensatory behaviours over the previous 7 days). The SEED demonstrates moderate construct validity, good criterion validity, and good sensitivity to change [48].

#### Primary outcome measure

##### Disordered eating behaviours and attitudes

The Eating Disorder Examination Questionnaire (EDE-Q; [49]) is a 28-item questionnaire assessing core attitudes and symptoms of disordered eating with four subscales: Dietary Restraint, Weight Concern, Shape Concern, and Eating Concern. The questionnaire spans a 28-day timeframe and is scored on a seven-point Likert scale ranging from 0 (*no days* or *not at* all) to 6 (*every day* or *markedly*). Higher scores indicate greater eating disorder psychopathology. The EDE-Q demonstrates good internal consistency and test–retest reliability [50–52].

#### Secondary outcome measures

##### Help-seeking, psychological and related measures

Secondary outcomes included help-seeking barriers, attitudes, intentions, and behaviours, body image, quality of life, social support, loneliness, self-esteem, depression and anxiety, and stages of change assessed with validated questionnaires (the specific scales employed are detailed in Additional file 1). Perceived need was assessed with the item “Do you believe you need help for eating, weight and shape concerns?” which was devised for the purposes of the trial.

##### Satisfaction with *ProYouth OZ* and evaluation of chat sessions

Satisfaction with the intervention was examined using 10 self-report items that have been used previously in trials of online interventions [53, 54]. In addition, participants were emailed a link after each chat session to indicate how satisfied they were with the session and whether they felt safe, informed, interested, supported, encouraged, and a sense of belonging. Participants also answered the following question: “Overall how satisfied were you with the chat session today?” with responses ranging from 1 (*not satisfied at all*) to 6 (*very satisfied*).

##### Program adherence

Adherence (i.e., user engagement with the program) was measured based on number of logins to the program, self-monitoring access, and chat session attendance. Given the unstructured nature of *ProYouth OZ*, we defined minimal usage as the following: (1) at least one login to the program, or (2) at least one completed self-monitoring.

### Data analysis

Given the high number of participants who failed to complete the post and follow-up assessments which resulted in low statistical power, it was not possible to conduct the initially planned analyses (details can be found at ACTRN12615001250527).

Participant flow was analysed in detail using descriptive and inferential statistics. Fisher’s exact test for categorical variables and one-way ANOVAs for continuous variables were used to compare pre-intervention characteristics and trial completion rates of the allocated groups, as well as eating disorder symptoms and/or demographic status as a function of eligibility at screening, recruitment modality, pre-intervention and assessment completion. One-way ANOVAs were conducted to compare adherence between intervention groups with adherence defined as at least one login and/or one completed self-monitoring. The number of program logins, the number of times participants viewed a web page of the program, and completed monitoring assessments were also compared in the two intervention groups using Mann–Whitney U tests. Finally, individual outcome profiles were constructed for participants from the intervention groups across measurement occasions.

## Results

**Fig. 1:**
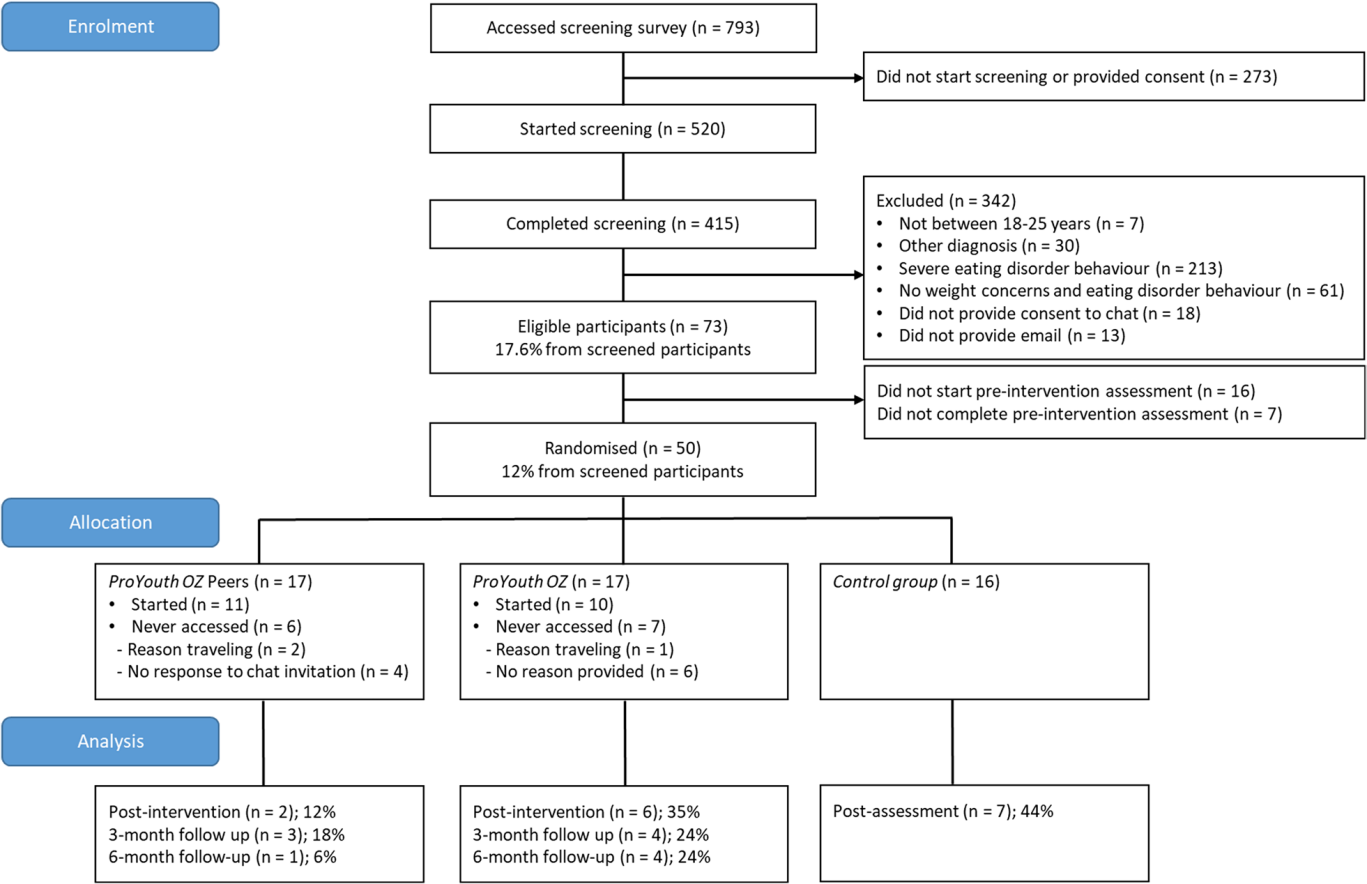
CONSORT: flow of participants

As shown in the CONSORT diagram (Fig. 1), almost 800 individuals showed interest in the study by accessing the screening survey, while more than half completed the screening (56.2%, *n* = 415). However, only a minority of screened participants were eligible (17.6%, *n* = 73), of whom 50 (12% of the screened sample) completed the pre-intervention assessment. There were substantial missing data at the post-intervention and follow-up assessments: only 15 (30%) of the 50 participants who were randomised completed a post-intervention assessment. There were no statistically significant differences in assessment completion rates between the three groups; Fisher’s exact test, *χ*^2^ (2) = 4.36, *p* = 0.113. Only seven (20.6%) and five participants (14.7%) from the two intervention groups combined (*ProYouth OZ* and *ProYouth OZ Peers*) completed the 3- and 6-month follow-up assessments, respectively. Completion rates for the two groups were not statistically significant different at either time point; Fisher’s exact tests, *χ*^*2*^ (1) = 0.07, *p* = 1.00 and *χ*^2^ (1) = 0.25, *p* = 0.335.

### Screening

#### Recruitment strategies

More than half of the participants (*n* = 223, 53.7%) who completed the screening were recruited via social media (e.g., Facebook, YouTube, etc.) or other online platforms (e.g., Google, mental health organisations such as the National Eating Disorders Collaboration or Butterfly Foundation); the remaining participants were recruited at universities (e.g., flyer, poster; *n* = 149, 35.9%), from friends (*n* = 13, 3.1%), via traditional media (e.g., newspaper, radio; *n* = 9, 2.2%) or clinical referrals (*n* = 11, 2.7%). Participants recruited online reported significantly greater weight concerns (WCS mean difference = 5.6; *t*(410) = 2.83, *p* = 0.005) compared to those recruited offline. In addition, a greater percentage of the participants recruited online reported vomiting (26.5% vs. 17.5% recruited offline; *χ*^2^ (1) = 4.78, *p* = 0.029), the misuse of laxatives (16.6% vs. 9.5% recruited offline; *χ*^2^ (1) = 4.42, *p* = 0.036), and current eating disorder diagnoses (75.3% vs. 58.6% recruited offline; *χ*^2^ (1) = 15.75, *p* < 0.001). There were no statistically significant differences for binge eating between those recruited online or offline.

#### Eligibility for study participation

Of the 415 participants who completed the screening, more than half (*n* = 213; 51.3%) were excluded due to the severity of their eating disorder symptoms (*n* = 112) or a current eating disorder diagnosis (*n* = 101). Another 14.7% of participants (*n* = 61) without weight or shape concerns and/or disordered eating behaviour were excluded. Further, some participants did not provide informed consent or a valid email address (*n* = 31, 7.5%). In summary, only 17.6% of screened individuals (*n* = 73) were eligible to participate, of whom another 23 (31.5% of eligible participants) did not start or finish the relevant measures of the pre-intervention assessment.

### Pre-intervention characteristics

Of the 73 eligible participants, only 50 completed the pre-intervention assessment. Table 1 presents demographic characteristics and eating disorder symptoms of eligible participants who either completed or failed to complete the pre-intervention assessment. There were no significant differences between those two groups.

**Table 1:** Comparison of demographics and eating disorder symptoms of eligible participants as a function of pre-intervention assessment completion

	Failed to complete assessment *n* = 23	Completed assessment *n* = 50	*p*
Age (*M, SD*)	22.2 (1.7)	21.3 (2.4)	0.053
Gender (female, *N*, %)^a^	19 (82.6)	47 (94)	0.078
Body mass index (*M, SD*)	23.4 (4.1)	24.1 (4.5)	0.516
Weight concern scale (*M, SD*)	69.9 (15.8)	70.4 (16.3)	0.901
Binge eating (*N*, %)^b^	14 (60.9)	28 (56)	0.801
Vomiting (*N*, %)^b^	3 (13)	3 (6)	0.371
Use of laxatives (*N*, %)^b^	2 (8.7)	3 (6)	0.647
Excessive exercise (*N*, %)^b^	14 (60.9)	23 (46)	0.315
Dieting (*N*, %)^b^	18 (78.3)	43 (86)	0.500

^a^Gender comparison was based on the male and female subgroups only, due to the small sample size of the “other” category size

^b^Frequency of binge eating, vomiting, use of laxatives, excessive exercise, dieting at least “less than once a week”

There was no association between recruitment modality and pre-intervention assessment completion (*χ*^2^ (1) = 0.08, *p* = 0.779), with 69.8% (*n* = 30) of the eligible offline-recruited participants completing the pre-intervention assessment compared to 66.7% (*n* = 20) of the online-recruited participants. Sociodemographic characteristics and baseline eating disorder symptoms of participants who were randomised are presented in Table 2. No significant differences in any of the sociodemographic variables were found between the intervention and control groups.

**Table 2:** Sociodemographic characteristics of randomised participants

	Total *N* = 50	*ProYouth OZ Peers n* = 17	*ProYouth OZ n* = 17	Control *n* = 16	*p*
Age (years; *M, SD*)	21.3 (2.4)	21.6 (2.2)	20.6 (2)	21.5 (2.9)	0.422
Gender^a^ (female; *N*, %)	47 (95.9)	16 (94.1)	15 (93.8)	16 (100)	0.602
Education (*N*, %)					0.774
Degree	29 (58)	11 (64.7)	9 (52.9)	9 (56.3)	
High school	21 (42)	6 (35.3)	8 (47.1)	7 (43.8)	
Employment status (N, %)					0.830
Student	33 (66)	13 (76.5)	10 (58.8)	10 (62.5)	
Working	14 (28)	3 (17.6)	6 (35.3)	5 (31.3)	
Other^b^	3 (6)	1 (5.9)	1 (5.9)	1 (6.3)	
Ethnicity (*N*, %)					0.605
Caucasian	40 (80)	14 (82.4)	13 (76.5)	13 (81.3)	
Asian	9 (18)	3 (17.6)	4 (23.5)	2 (12.5)	
Other^c^	1 (2)	–	–	1 (6.3)	

^a^Gender comparison was based on male vs. female subgroups only, due to the small sample size of the “other” category

^b^Other employment status comprising unemployed, home duties, not working due to illness, volunteer work

^c^Other ethnicity comprising Aboriginal/Torres Strait Islander, African, other

### Engagement

#### Intervention access and minimal intended use

Of the 34 participants who were randomised to one of the two intervention groups, seven (20.6%) failed to access any component of *ProYouth OZ* over the 6-week intervention period. There were no statistically significant differences in eating disorder severity or the perceived need for help between participants who did and did not access the intervention (*p* > 0.150).

#### Logins and page hits

Of the 34 participants who were randomised to one of the two intervention groups, 13 (38.2%) failed to log in to the program at all (three participants due to overseas travel plans during the trial, 10 for reasons unknown), whereas 21 participants logged in to the program at least once (see Table 3). The number of logins ranged from 1 to 17 (*Median* = 3), with five participants accessing the program only once of whom one was from the *ProYouth OZ* Peer group and four were from the *ProYouth OZ* group. Six participants accessed the program more than five times, including five participants from the *ProYouth OZ* Peer group and one participant from the *ProYouth OZ* group. Although participants from the *ProYouth OZ* Peer group appeared to access the program more frequently compared to the *ProYouth OZ* group, there were no statistically significant differences in login frequencies across the two intervention groups (Mann–Whitney U test, *U* = 31.50, *p* = 0.099).

**Table 3:** ProYouth OZ Usage (i.e., logins, page hits, monitoring assessments)

	Total (*n* = 23)	*ProYouth OZ* Peers (*n* = 11)	*ProYouth OZ* (*n* = 10)	Control group^#^ (*n* = 2)
Logins				
N (%)	97	61 (62.9%)	32 (33.0%)	4 (4.1%)
M (*SD*)	4.22 (4.13)	5.55 (4.61)	3.20 (3.68)	2.00 (1.41)
Md (*IQR*)	3 (5)	5 (5)	2 (3)	2
Page hits				
N (%)	403	208 (51.6%)	176 (43.7%)	19 (4.7%)
M (*SD*)	17.52 (18.57)	18.91 (15.35)	17.60 (23.71)	9.5 (4.95)
Md (*IQR*)	13 (11)	18 (18)	11.5 (15)	9.5

	Total	*ProYouth OZ* Peers	*ProYouth OZ*	Control group
	(*n* = 29)	(*n* = 11)	(*n* = 14)	(*n* = 4)
Monitoring				
M, SD	2.55 (1.88)	1.82 (1.54)	3.07 (1.77)	2.75 (2.87)
Md, IQR	2 (3)	1 (1)	3 (4)	1.5 (5)

Logins = number of logins to *ProYouth OZ*, Page hits = number of pages accessed in the participation area (i.e., after login), Monitoring = number of completed monitoring assessments, IQR = inter quartile range

^#^Control group participants were waitlisted to receive the intervention after the pre, post, and 3-month follow-up assessments

The total number of times participants viewed a web page of the program in the two *ProYouth OZ* intervention groups was 403, ranging from 3 to 83. On average, participants viewed 18.29 (*SD* = 19.26, *Median* = 13) individual web pages. There were no statistically significant differences in page hits between the two groups (Mann–Whitney U test, *U* = 43.50, *p* = 0.435).

#### Monitoring assessments

Of the 34 participants who were randomised to one of the two intervention groups, nine (26.5%) did not complete any monitoring assessments, whereas 25 participants completed at least one monitoring assessment. The number of weekly monitoring assessments ranged from 0 to 6. Some participants completed the weekly monitoring assessments (*n* = 6) without accessing the program or accessed the program without completing the monitoring (*n* = 2). Most participants completed only one monitoring (*n* = 11, 44%), whereas a minority completed all six assessments (*n* = 2, 8%). On average, participants from the two intervention groups completed 2.52 monitoring assessments (*SD* = 1.76, *Median* = 2), and the number of completed monitoring assessments did not significantly differ between the two intervention groups (Mann–Whitney U test, *U* = 44.50, *p* = 0.062). Amongst the 25 participants who completed at least one monitoring assessment, in total 63 assessments were completed. This equates to 42% of the six scheduled monitoring assessments for each participant.

#### Chat session attendance

Participants in the *ProYouth OZ Peers* condition were invited to attend six chat sessions (one chat session per week over 6 weeks). Of the 17 participants randomised to the condition, three participants were not able to attend any chat session on the scheduled dates, and one participant did not reply to the chat invitation email. In total, 13 (76.5%) were scheduled for weekly chat sessions; however, two participants informed the trial manager that they were not able to attend any sessions due to conflicting commitments (e.g., travelling), and another participant failed to attend but offered no explanation for their absence (see Additional file 2). In total, 10 (58.8%) of the randomised participants attended at least one chat session: nine participants attended the first session, five the second session, three attended the third and the fourth sessions, and two participants attended the final session. Only 20% attended more than two out of six sessions, and only two participants (11.8% of 17 eligible participants) attended all six sessions.

### Satisfaction with *ProYouth OZ*

The two participants who attended all chat sessions in *ProYouth OZ Peers* and completed the post-intervention assessment reported that they would use the program again in the future, would definitely recommend it to friends, and were overall very satisfied with their experience. Of the six participants in the *ProYouth OZ* group, 50% reported that they would use the program again and probably recommend it to friends. Overall, one participant was very satisfied, one participant was not satisfied with the program, and four participants were neither satisfied nor dissatisfied.

#### Satisfaction with chat sessions

All participants who attended chat sessions were invited to complete satisfaction ratings following each session. The overall satisfaction rating with the chat sessions was high (*M* = 5.00, *SD* = 1.24; *n* = 14). Furthermore, five participants provided open ended qualitative feedback about the sessions. Some provided overall positive feedback: “*It was good to articulate thoughts, very cathartic”, “… it was a great session…”, “It was interesting hearing the experience of someone who described themselves on the other end of the spectrum weight wise to my own experiences…”*. Some commended the friendly chat environment: “*Really enjoyed the welcoming atmosphere, I feel like I gained a lot from the discussion!”*, with a number specifically singling out the input of the facilitators as follows: “*Both facilitators were fantastic to talk to! Such a supportive and safe environment, the discussion was always interesting and relatable, and I felt like I gained a lot of insight from the discussions over the weeks”; and “I feel safe knowing the facilitator is listening to me and actively trying to support me—the facilitator is a wonderful moderator and always very encouraging!”*. Others highlighted the use of videos: “*I really like ProYouth OZ's approach of using videos from people who have experienced eating disorders before, as it gave me a way of feeling actually personally connected to someone in my own body issue struggles.”.*

However, the low participation rate was seen as a limitation by some: “*It would have been good to get more out of the other participant as I did feel like a lot of the time I contributed most…”; “It was just me talking so I didn't really get to bounce ideas and thoughts off of others, but it was very nice to talk to the facilitator and be able to focus on my own particular body image issues”*; and “*It was just the facilitator and I, it would be nice to have someone to share experiences with…”.* In addition, one participant suggested restructuring the chat sessions: “*We had to run over time in our chat (which was great) but it would be even better if the next chats just launched into the discussion straight away…*”. Finally, one participant reported that the program did not meet her needs: “*This course is not designed or supportive of people who are also battling real weight issues, another resource aimed only at skinny people with eating disorders*”.

### Primary outcomes: eating disorder severity

**Fig. 2:**
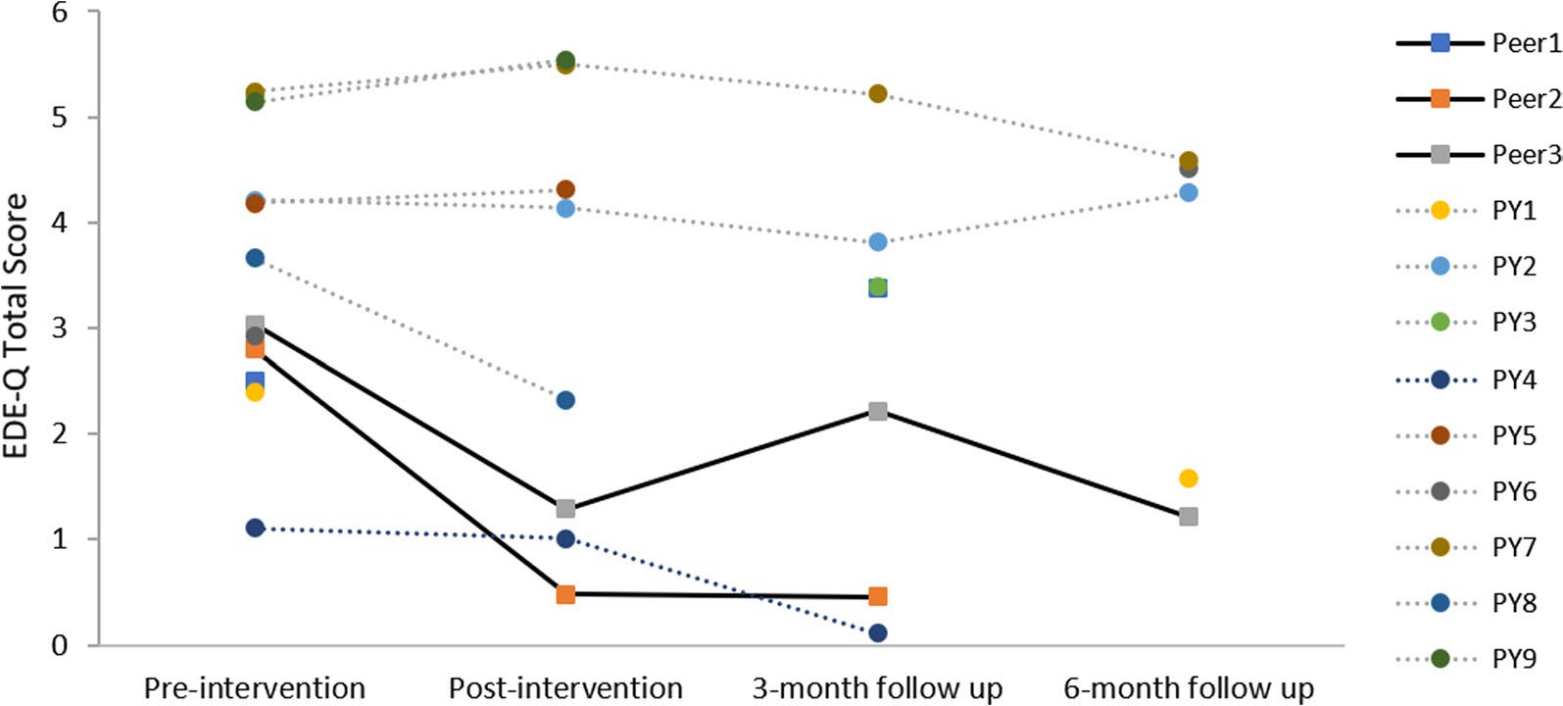
Eating Disorder Examination Questionnaire Total Score Profiles of ProYouth OZ and ProYouth OZ Peers Participants who completed at least one post-intervention assessment

Table 4 presents observed means and standard deviations for the primary outcome variables (i.e., EDE-Q total and subscales, objective binge eating, vomiting, use of laxatives) at the four measurement occasions for each condition. We also present individual EDE-Q profiles of the participants from the two intervention groups who provided post-intervention data (see Fig. 2 and Additional file 3). Visual inspection indicates that both participants from *ProYouth OZ Peers* showed decreased EDE-Q scores at post assessment. Conversely, only one of the six participants in *ProYouth OZ* (PY4 in Fig. 2) reported a decreased EDE-Q total score at the post assessment point.

**Table 4:** Observed means and standard deviations for EDE-Q total and subscales scores and frequencies of eating disorder behaviours (objective binge eating, vomiting, use of laxatives) across different assessment points

	Pre-intervention					Post-intervention			3-month follow up		6-month follow up	
	Total *N* = 50	PY Peers *n* = 17	PY *n* = 17	Control *n* = 16	*p*^a^	PY Peers *n* = 2	PY *n* = 6	Control *n* = 7	PY Peers *n* = 3	PY *n* = 4	PY Peers *n* = 1	PY *n* = 4
EDE-Q Total (*M, SD*)	3.28 (1.02)	3.35 (0.83)	3.38 (1.32)	3.10 (0.88)	0.708	0.89 (0.57)	3.80 (1.80)	3.38 (1.59)	2.02 (1.47)	3.13 (2.16)	1.21	3.74 (1.45)
Restraint (*M, SD*)	2.94 (1.42)	2.80 (1.22)	3.05 (1.60)	2.96 (1.42)	0.871	0.40 (0.57)	3.57 (1.89)	3.63 (1.97)	0.87 (0.90)	3.10 (2.20)	0.60	3.40 (1.90)
Eating Concern (*M, SD*)	2.16 (1.14)	2.33 (1.00)	2.08 (1.47)	2.06 (0.89)	0.758	0.40 (0.57)	3.17 (1.89)	2.17 (1.66)	1.07 (1.36)	2.15 (1.84)	0.60	2.75 (1.34)
Shape Concern (*M, SD*)	4.28 (1.00)	4.43 (0.76)	4.49 (1.61)	3.91 (1.01)	0.192	1.25 (0.18)	4.48 (1.79)	3.98 (1.73)	3.08 (1.95)	3.94 (2.53)	1.63	4.56 (1.38)
Weight Concern (*M, SD*)	3.75 (1.19)	3.85 (1.12)	3.89 (1.42)	3.49 (1.02)	0.577	1.50 (0.99)	4.00 (1.93)	3.74 (1.37)	3.07 (2.25)	3.35 (2.33)	2.00	4.25 (1.24)
OBE (*N*, %)					0.341							
No OBE	15 (30)	3 (17.6)	7 (41.2)	5 (31.3)		1 (50)	1 (16.7)	1 (14.3)	2 (66.7)	3 (75)	1 (100)	1 (25)
OBE 1–3	14 (28)	5 (29.4)	6 (35.3)	3 (18.8)		1 (50)	1 (16.7)	2 (28.6)	0	0	0	2 (50)
OBE ≥ 4	21 (42)	9 (52.9)	4 (23.5)	8 (50)		0	4 (66.7)	4 (57.1)	1 (33.3)	1 (25)	0	1 (25)
Vomiting (*N*, %)					0.897							
No vomiting	45 (90)	16 (94.1)	15 (88.2)	14 (87.5)		2 (100)	4 (66.7)	7 (86.7)	3 (100)	4 (100)	1 (100)	3 (75)
1–3 vomiting	3 (6)	1 (5.9)	1 (5.9)	1 (6.3)		0	1 (16.7)	1 (6.7)	0	0	0	1 (25)
≥ 4 vomiting	2 (4)	0	1 (5.9)	1 (6.3.)		0	1(16.7)	1 (6.7)	0	0	0	0
Use of laxatives (*N*, %)					0.380							
No use of lax	46 (92)	17 (100)	14 (82.4)	15 (93.8)		2 (100)	5 (83.5)	6 (85.7)	3 (100)	4 (100)	1 (100)	2 (50)
1–3 use of lax	3 (6)	0	2 (11.8)	1 (6.3)		0	0	1 (6.7)	0	0	0	0
≥ 4 use of lax	1 (2)	0	1 (5.9)	0		0	1 (16.7)	0	0	0	0	2 (50)

*EDE-Q* eating disorders examination-questionnaire, *OBE* objective binge eating

^a^One-way ANOVAs for continuous variables, Fisher’s exact test for categorical variables

## Discussion

This study aimed to investigate the effectiveness of *ProYouth OZ*, an indicated Internet-based prevention program for young adults at risk of eating disorders. Although it was not possible to conduct group comparisons, nor to test for any pre- to post-intervention changes between the groups over time due to the small sample size and resulting lack of power, we did examine the individual outcome profiles of participants in each intervention condition for whom pre- and post-intervention data were available. Whereas most of the participants in the *ProYouth OZ* condition (i.e., 5 out of 6) failed to show any change in symptoms, visual inspection of the data showed that both participants in the *ProYouth OZ Peers* condition reported decreased eating disorder symptoms immediately after the intervention. This pattern of finding is consistent with Kass et al.’s [32] result for asynchronous peer support and suggests that the role of synchronous peer support warrants further investigation. The discussion below focuses on the lessons learned from this trial that may inform the design of such studies.

### Challenges and lessons learned

Recruitment challenges, low adherence, and high rates of attrition commonly compromise randomised controlled trials [55, 56] and in this respect our results are not unique. Thus, it has been argued that it is important to report “failed” trials as well as trials with negative or null results [57, 58].

#### Recruitment and screening of eligible participants

The current study sought to include individuals with elevated weight and shape concerns and/or subthreshold eating disorder symptoms and exclude those with a diagnosable eating disorder. The majority of interested individuals (51%) were excluded due to their severe eating disorder pathology, in marked contrast to less than 15% in other indicated prevention trials [25, 27, 29]. It is likely that this difference is attributable at least in part to extending recruitment for the current study to websites of eating disorder organisations and mental health services. Despite the use of the same advertisement text in the online and offline recruitment strategies, interested participants from online settings reported significantly higher weight and shape concerns and rates of eating disorder diagnosis than those from offline strategies. Bauer et al. [59] reported a similar trend, arguing that participants recruited online are subject to greater self-selection. Thus, it may be that individuals who showed interest in *ProYouth OZ* online were more actively seeking eating disorder related information and support. Importantly, while this may have resulted in a high level of exclusion in the current study due to symptom severity, online recruitment nevertheless attracted the majority of eligible participants (60%), a finding of potential relevance when designing prevention trials.

High rates of ineligibility due to severe symptoms may also have been partially due to our screening method. Other trials of online programs employed either face-to-face or telephone diagnostic interviews [25, 28] or an online validated structured self-report measure [29] to exclude participants with an eating disorder. It is possible that due to the use of a brief self-report screener, the present study may have overdiagnosed eating disorders. Nevertheless, based on the current and previous research, it is likely that indicated Internet-based intervention outcome studies require large numbers of potential participants to be screened to achieve samples that meet certain eligibility criteria (i.e., 1500–4500 participants) [25, 28, 29]. Future studies should report details on recruitment pathways and advertising materials, screening processes, and associations between recruitment strategies and eligibility to identify the recruitment strategies that most successfully attract eligible participants for indicated prevention programs.

As noted above, although also employed in other indicated eating disorder prevention trials [25, 26, 29] the online screening approach used in the current study is not without its limitations. Telephone or face-to-face interview processes [25–27] have the advantage of accurately excluding those with full syndrome eating disorders and may increase participant commitment. However, the use of face-to-face assessments mitigates some of the key benefits of Internet-based interventions including accessibility, anonymity, and relatively low intervention cost. More importantly, person-to-person assessments are less feasible in real-world settings when the aim is to broadly disseminate programs to a wider population. Thus, a focus on refining online screening is warranted. For example, the development of inclusion and exclusion criteria to identify participants at risk is complex and has been applied differently across studies. While the current study used a combination of moderate to high weight and shape concerns (i.e., WCS > 57) as well as dysfunctional eating behaviours to include participants, which may have contributed to the low number of eligible individuals, other studies of indicated prevention programs have broadened their criteria to include individuals with lower levels of weight concerns (e.g., WCS > 47; [25, 26]) or body dissatisfaction only without any additional behavioural criteria [29]. It has been suggested that highly specific inclusion criteria may result in difficulties identifying suitable participants, especially for low prevalence conditions and hard-to-reach populations [55]. There is therefore an urgent need for further research on online screening algorithms to determine which eligibility criteria result in an at-risk sample that is most likely to access and benefit from these programs.

#### Program engagement

The finding that 21% of the intervention participants failed to log in or complete a monitoring assessment is consistent with other indicated prevention trials of the more structured programs *StudentBodies* and *The Body Project* where 5–19% of participants never accessed the intervention [26–29]. Over 73% of participants from the two intervention groups completed at least one self-monitoring and participants completed 42% of all six scheduled monitoring assessments. Low adherence is not surprising considering the unstructured nature of *ProYouth OZ*. However, in the four indicated trials of *StudentBodies,* participants completed between 12 and 68% of required monitoring assessments [25–28], suggesting that adherence to more structured interventions are not invariably superior. It may be that participants in the current trial did not perceive self-monitoring to be useful, beneficial or engaging, and accordingly ceased using this component of the program. Consistent with the latter interpretation, results from the *ProYouth* program in Germany suggest that participants prefer to use more interactive components of the intervention [36, 59], such as the forum or chat sessions [60].

Given that the study was designed to examine the impact of online peer support, engagement with the chat sessions was of particular interest. Although almost 60% of participants from the *ProYouth OZ Peers* group attended at least one chat session, engagement decreased over time with only 20% of the participants attending more than two of the six scheduled sessions. It is noteworthy that participants who attended chat sessions were generally satisfied with this program component, with qualitative feedback indicating that they valued the safe and supportive environment, the interesting and relatable content, and the benefits gained from the discussion with others who were experiencing similar problems. Some participants particularly highlighted the positive interaction with the peer facilitator and the moderator.

In the current trial, no associations were found between program completion and perceived need for help or severity of eating disorder symptoms. This is inconsistent with studies of other programs, such as the *Healthy Body Image* program [61] and *Student Bodies* [28], which found that higher adherence was associated with higher perceived need for help and higher eating pathology, respectively. However, other findings with regard to the relationship between symptom severity and program engagement are mixed [26, 29]. These inconsistent findings raise the possibility that different programs are suitable for different individuals along the continuum of eating disorder symptoms and is a question that requires further investigation.

Although Internet-based programs can overcome some of the practical barriers of face-to-face preventive approaches (e.g., flexible delivery independent of time and place), the time required to participate in these programs can constitute a substantial barrier [62]. For example, in the current trial, some participants reported that they were unable to attend chat sessions due to other commitments. On the other hand, feedback from *ProYouth OZ* Peers positively highlighted the helpfulness of peer videos in recovery. These findings suggest that young adults may respond better to brief, engaging, visual (as opposed to text) content that they can use independent of specifically scheduled sessions.

#### Assessment completion

Failure to complete assessments has been reported as a common issue in Internet-based interventions [63]. Assessment completion in the current trial was low, with only 30% of participants completing post-intervention measures (i.e., 24% from the intervention groups vs. 44% from the control group). Thus, attrition rates was higher compared to indicated trials of *StudentBodies* and *The Body Project*, where assessment completion ranged from 63 to 91% at post-intervention [25–27, 29]. Notably, most of the latter trials assessed participants face-to-face or on the telephone at all time points and the lack of personal contact with the research team in the current trial may also have negatively impacted on assessment completion.

Further, in other studies with higher completion rates, participants were often provided with incentives for completing study assessments [27, 29]. Monetary incentives have been found to increase enrolment and assessment completion in online interventions [64, 65]. In fact, a previous meta-analysis of the findings from *StudentBodies* has highlighted that the provision of incentives may be essential for assessment completion [66]. In contrast, in order to model real-world conditions, the current study did not offer monetary incentives for completing assessments. The absence of such payments in combination with the lack of face-to-face assessments may account for the low completion of assessments [67]. Importantly, however, these expensive strategies raise questions about the external validity of findings.

It may be that other factors including the timing or length of assessments (duration of 30 min) further impacted completion rates. A less ambitious set of aims and a corresponding reduction in outcome measures might have increased completion rates and the ecological validity of the study given that such outcomes would not normally be incorporated into a real-world intervention.

### Future research and implications

Although no conclusions can be drawn about the effectiveness of *ProYouth OZ* and the additional effect of online peer support, findings from the current study have important implications for future developments and research. Consistent with existing evidence, our findings demonstrate that young adults at risk for eating disorders are a difficult to reach and engage population [28, 59]. Efficacious prevention programs will not benefit individuals in real-world settings if they fail to reach the broader target populations for whom the programs were designed. Given that prevention programs have maximal potential to reduce disease burden [68], it is essential to identify the most effective strategies to reach and engage these individuals. Thus, future studies should extend emerging research to identify optimal strategies for reaching at-risk populations (e.g., online vs offline) as this will inform important future dissemination efforts [28, 59]. As argued previously [24], there is also an urgent need to understand who and under what conditions young people engage or fail to engage in Internet-based prevention programs for eating disorders. For example, future research should systematically measure baseline perceived need and motivation to change to better understand if and how these factors impact uptake and engagement. Future research should also investigate if, as proposed by Wilksch et al. [69], brief motivation enhancement techniques pre-intervention increase the likelihood of participants engaging with eating disorder prevention programs.

There is also a need to examine other potential interventions for increasing engagement such as the use of co-design [70] and involving peers in the promotion of such programs in order to enhance a sense of belonging and increase perceived need for the intervention. Since videos of young adults with a lived experience were regarded positively by participants in the current trial, future studies could examine the potential of peer support to increase engagement with the intervention. The use of gamification in such programs might also impact on engagement in young people at risk of eating disorder [71, 72], especially as individuals with these disorders are often highly perfectionistic and achievement focussed [73, 74]. In the broader field of behavioural economics, financial incentives have been found to be a useful strategy to promote healthy and discourage unhealthy behaviours. A recent conceptual framework of engagement with digital behaviour change interventions hypothesised an association between rewards, motivation, and engagement [75, 76]. Therefore, it may be worthwhile to implement incentives in real-world settings since reducing unhealthy disordered eating behaviour is highly desirable from a public policy perspective. However, carefully designed cost–benefit studies would need to establish the sustainability of such approaches.

A final consideration concerns the nature of the unmet need in the community (as demonstrated by the high number of interested individuals in the current trial) as it relates to the programs offered. While online recruitment in the current study appeared to attract more participants with severe eating disorder symptoms, *ProYouth OZ* was not designed for those with a diagnosable disorder. In fact, the majority of Internet-based programs for eating disorders were developed as prevention programs (universal, selective, or indicated) rather than treatment programs. There may be a need to develop new and innovative interventions designed specifically for individuals with eating disorders who choose to access help online but may not be ready or able to seek professional face-to-face treatment due to a variety of barriers. Such programs could provide initial self-help strategies and information designed to facilitate help-seeking, to reduce the significant treatment gap in the field of eating disorders and consequently reduce the disease burden [68].

## Conclusion

Although limited, the outcome data in the current study suggests that future research on peer-to-peer support is warranted. Individuals at-risk for eating disorders are difficult to reach and future trial protocols must incorporate large-scale screening strategies to recruit a sufficient number of eligible individuals. Challenges related to adherence and attrition highlighted the importance of systematically investigating for whom and under which conditions Internet-based prevention programs for eating disorders work and evaluating the effectiveness of a range of strategies for promoting engagement. Finally, the study pointed to the large group of interested young adults with high levels of unmet need who, although ineligible for an indicated prevention trial due to their elevated symptomatology, respond to simple recruitment advertisements and might benefit from tailored resources that facilitate their access to face-to-face treatment.

## Supplementary Information

**Additional file 1:** Measures Used at Screening, Pre- and Post-intervention and Follow-ups (FU).

**Additional file 2:** Attendance of ProYouth OZ Peers Chat Sessions.

**Additional file 3: **Eating Disorder Examination Questionnaire Subscale Score (Restraint, Eating Concern, Shape Concern, Weight Concern) Profiles of ProYouth OZ Peers and ProYouth OZ Participants at Pre- and Post-intervention and 3- and 6-months Follow-ups.

## Data Availability

The data that support the findings of this study are available from the corresponding author upon reasonable request.
