# Supplementary material for: Recruitment, adherence and attrition challenges in internet-based indicated prevention programs for eating disorders: lessons learned from a randomised controlled trial of ProYouth OZ
Source: J Eat Disord. 2022 Jan 4;10:1. doi: 10.1186/s40337-021-00520-7 (PMC8725518; doi:10.1186/s40337-021-00520-7)
Supplement: Supplementary file 1 — Additional file 1: Measures Used at Screening, Pre- and Post-intervention and Follow-ups (FU). [file 40337_2021_520_MOESM1_ESM.docx]

**Additional file 1.**

*Measures Used at Screening, Pre- and Post-intervention and Follow-ups (FU)*

| Assessment | Screening | Monitoring | Pre | Post | FU (3-month) | FU (6-month) |
| --- | --- | --- | --- | --- | --- | --- |
| Weight Concerns Scale (WCS) (1, 2) | x |  | x | x | x | x |
| Short Evaluation of Eating Disorders (SEED) (3) | x |  | x | x | x | x |
| Monitoring questionnaire |  | x |  |  |  |  |
| Sociodemographics |  |  | x |  |  |  |
| Eating Disorder Examination Questionnaire (EDE-Q) (4) |  |  | x | x | x | x |
| Body Shape Questionnaire-Short Form (BSQ-SF) (5) |  |  | x | x | x | x |
| Sociocultural Attitudes Towards Appearance Scale (SATAQ-4) (6, 7) |  |  | x | x | x | x |
| Eating Disorder Literacy (EDL) |  |  | x | x | x | x |
| Eating Disorder Stigma Scale (USS) |  |  | x | x | x | x |
| Barriers Towards Seeking Help for Eating Disorders (BATSH-ED) (8) |  |  | x | x | x | x |
| Attitudes Toward Seeking Professional Help (ATSPPH) (9) |  |  | x | x | x | x |
| General Help Seeking Questionnaire (GHSQ) (10) |  |  | x | x | x | x |
| Actual Help Seeking Questionnaire (AHSQ) (10) |  |  | x | x | x | x |
| Quality of Life (EURhisQoL-8) (11) |  |  | x | x | x | x |
| Social Support (MOS-SS) (12) |  |  | x | x | x | x |
| Loneliness (UCLA) (13) |  |  | x | x | x | x |
| Rosenberg Self-Esteem Scale (RSE) (14) |  |  | x | x | x | x |
| Patient Health Questionnaire 4 (PHQ-4) (15) |  |  | x | x | x | x |
| Stages of Change Questionnaire (SOCQ-ED) (16, 17) |  |  | x | x | x | x |
| Empowerment (18) |  |  |  | x |  |  |
| Satisfaction with Intervention Questionnaire |  |  |  | x |  |  |
| Chat Group Evaluation |  |  |  | x |  |  |

**Reference**

1. Killen JD, Taylor CB, Hayward C, Haydel KF, Wilson DM, Hammer L, et al. Weight concerns influence the development of eating disorders: A 4-year prospective study. Journal of Consulting and Clinical Psychology. 1996;64(5):936-40.

2. Killen JD, Taylor CB, Hayward C, Wilson DM, Haydel KF, Hammer LD, et al. Pursuit of thinness and onset of eating disorder symptoms in a community sample of adolescent girls: A three-year prospective analysis. International Journal of Eating Disorders. 1994;16(3):227-38.

3. Bauer S, Winn S, Schmidt U, Kordy H. Construction, scoring and validation of the Short Evaluation of Eating Disorders (SEED). European Eating Disorders Review. 2005;13(3):191-200.

4. Fairburn CG, Beglin SJ. Assessment of eating disorders: Interview or self-report questionnaire? International Journal of Eating Disorders. 1994;16(4):363-70.

5. Cooper PJ, Taylor MJ, Cooper Z, Fairbum CG. The development and validation of the body shape questionnaire. International Journal of Eating Disorders. 1987;6(4):485-94.

6. Schaefer LM, Burke NL, Thompson JK, Dedrick RF, Heinberg LJ, Calogero RM, et al. Development and validation of the Sociocultural Attitudes Towards Appearance Questionnaire-4 (SATAQ-4). Psychol Assess. 2015;27(1):54-67.

7. Thompson JK, van den Berg P, Roehrig M, Guarda AS, Heinberg LJ. The sociocultural attitudes towards appearance scale-3 (SATAQ-3): development and validation. Int J Eat Disord. 2004;35(3):293-304.

8. Ali K, Fassnacht DB, Farrer L, Rieger E, Feldhege J, Moessner M, et al. What prevents young adults from seeking help? Barriers toward help-seeking for eating disorder symptomatology. International Journal of Eating Disorders. 2020;n/a(n/a).

9. Elhai JD, Schweinle W, Anderson SM. Reliability and validity of the Attitudes Toward Seeking Professional Psychological Help Scale-Short Form. Psychiatry Res. 2008;159(3):320-9.

10. Rickwood D, Deane FP, Wilson CJ, Ciarrochi J. Young people's help-seeking for mental health problems. AeJAMH (Australian e-Journal for the Advancement of Mental Health). 2005;4(3):No Pagination Specified-No Pagination Specified.

11. Schmidt S, Muehlan H, Power M. The EUROHIS-QOL 8-item index: Psychometric results of a cross-cultural field study. European journal of public health. 2006;16:420-8.

12. Sherbourne CD, Stewart AL. The MOS social support survey. Social Science & Medicine. 1991;32(6):705-14.

13. Russell DW. UCLA Loneliness Scale (Version 3): reliability, validity, and factor structure. J Pers Assess. 1996;66(1):20-40.

14. Rosenberg M. Society and the adolescent self-image, Rev. ed. Middletown, CT, England: Wesleyan University Press; 1989. xxxii, 347-xxxii, p.

15. Kroenke K, Spitzer RL, Williams JB, Lowe B. An ultra-brief screening scale for anxiety and depression: the PHQ-4. Psychosomatics. 2009;50(6):613-21.

16. Rieger E, Touyz SW, Beumont PJ. The Anorexia Nervosa Stages of Change Questionnaire (ANSOCQ): information regarding its psychometric properties. Int J Eat Disord. 2002;32(1):24-38.

17. von Brachel R, Hötzel K, Schloßmacher L, Hechler T, Kosfelder J, Rieger E, et al. Entwicklung und Validierung einer deutschsprachigen Skala zur Erfassung der Veränderungsmotivation bei Essstörungen – The Stages of Change Questionnaire for Eating Disorders (SOCQ-ED). Psychother Psych Med. 2012;62(12):450-5.

18. van Uden-Kraan CF, Drossaert CH, Taal E, Seydel ER, van de Laar MA. Participation in online patient support groups endorses patients' empowerment. Patient Educ Couns. 2009;74(1):61-9.
