# Supplementary material for: Recruitment, adherence and attrition challenges in internet-based indicated prevention programs for eating disorders: lessons learned from a randomised controlled trial of ProYouth OZ
Source: J Eat Disord. 2022 Jan 4;10:1. doi: 10.1186/s40337-021-00520-7 (PMC8725518; doi:10.1186/s40337-021-00520-7)
Supplement: Supplementary file 2 — Additional file 2: Attendance of ProYouth OZ Peers Chat Sessions. [file 40337_2021_520_MOESM2_ESM.docx]

**Additional file 2.**

*Attendance of ProYouth OZ Peers Chat Sessions*

| Chat session | Attendance (*n*, % of 17 eligible participants) | Reasons for not attending | Satisfaction  (M, SD) |
| --- | --- | --- | --- |
|  |  | *n* = 3 not available at agreed chat times  *n* = 2 could not attend due to conflicting commitments  *n* = 1 did not respond to chat scheduling email |  |
| 1 | 9 (52.9) | *n* = 2 did not attend, no reason provided | 5.0 (1.0);  *n* = 5 |
| 2 | 5 (29.4) | *n* = 2 sent email with apologies  *n* = 4 did not attend, no reason provided | 5.3 (1.2);  *n* = 3 |
| 3 | 3 (17.6) | *n* = 2 sent email with apologies  *n* = 6 did not attend, no reason provided | 4.7 (2.3);  *n* = 3 |
| 4 | 3 (17.6) | *n* = 1 sent email with apologies  *n* = 8 did not attend, no reason provided | 4.0; *n* = 1 |
| 5 | 2 (11.8) | *n* = 1 sent email with apologies  *n* = 8 did not attend, no reason provided | 6.0; *n* = 1 |
| 6 | 2 (11.8) | *n* = 1 sent email with apologies  *n* = 8 did not attend, no reason provided | 5.0; *n* = 1 |

*Note.* Satisfaction scale (1 = *not satisfied at all*; 6 = *very satisfied*).
