# Supplementary material for: Recruitment, adherence and attrition challenges in internet-based indicated prevention programs for eating disorders: lessons learned from a randomised controlled trial of ProYouth OZ
Source: J Eat Disord. 2022 Jan 4;10:1. doi: 10.1186/s40337-021-00520-7 (PMC8725518; doi:10.1186/s40337-021-00520-7)

**Additional file 3.**

*Eating Disorder Examination Questionnaire Subscale Score (Restraint, Eating Concern, Shape Concern, Weight Concern) Profiles of ProYouth OZ Peers and ProYouth OZ Participants at Pre- and Post-intervention and 3- and 6-months Follow-ups*


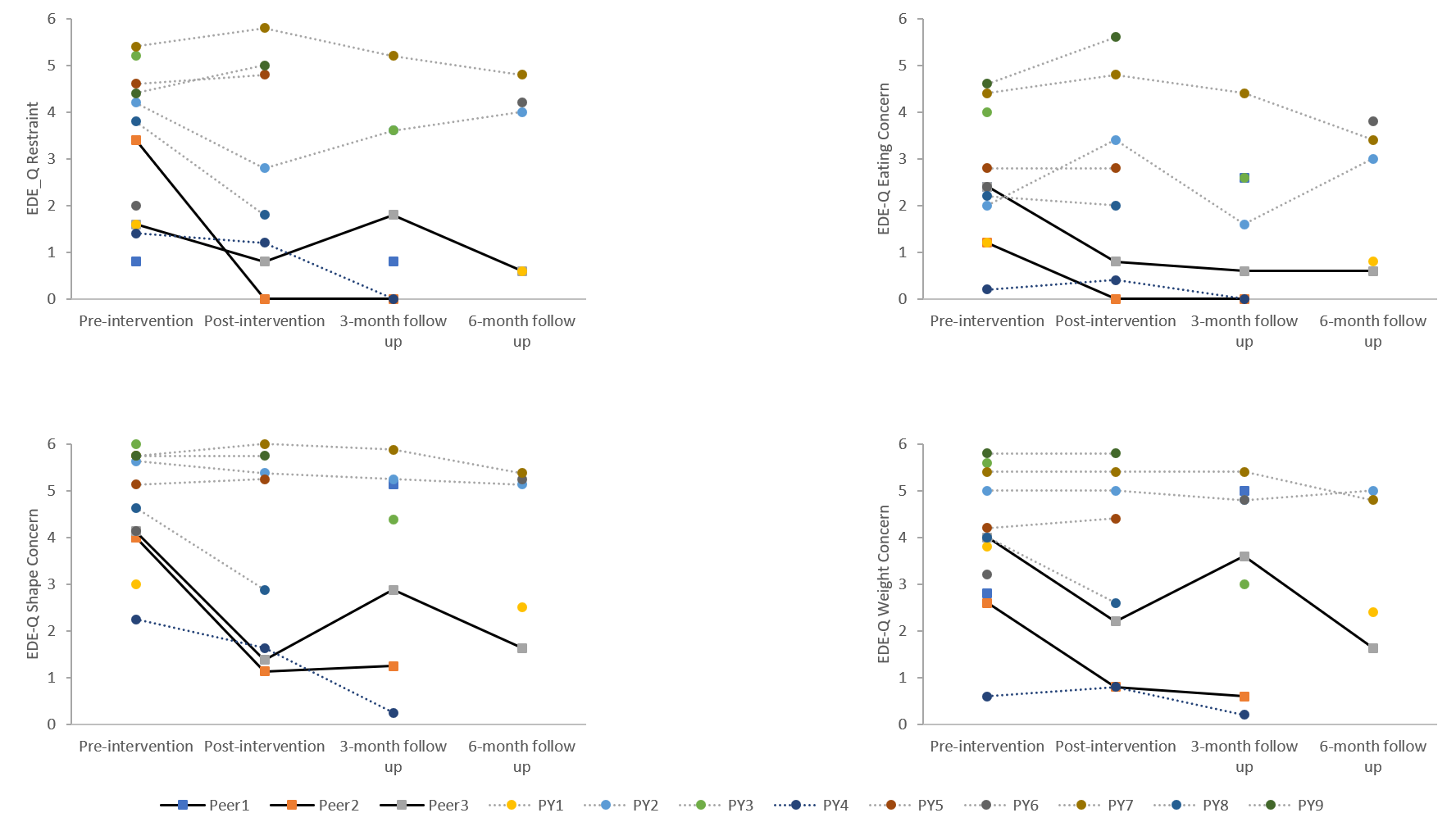

Supplement: Supplementary file 3 — Additional file 3: Eating Disorder Examination Questionnaire Subscale Score (Restraint, Eating Concern, Shape Concern, Weight Concern) Profiles of ProYouth OZ Peers and ProYouth OZ Participants at Pre- and Post-intervention and 3- and 6-months Follow-ups. [file 40337_2021_520_MOESM3_ESM.docx]
